# Supplementary material for: Causal Relationship between Aging and Anorexia Nervosa: A White-Matter-Microstructure-Mediated Mendelian Randomization Analysis
Source: Biomedicines. 2024 Aug 16;12(8):1874. doi: 10.3390/biomedicines12081874 (PMC11351342; doi:10.3390/biomedicines12081874)
Supplement: Supplementary file 1 [file biomedicines-12-01874-s001.zip › Supplementary Figure/Figure S8/Leaveoneout plot of MR analysis for TL to ubm-b-1687.pdf]

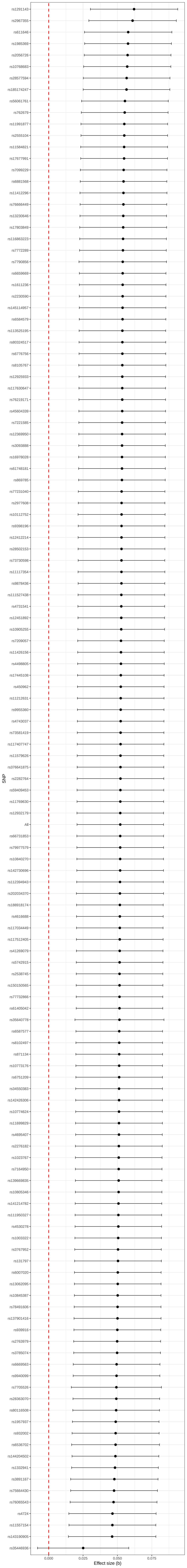
rs1291143
rs2967355
rs611646
rs1985369
rs2056726
rs10768683
rs28577594
rs185174247
rs56061761
rs762679
rs11991877
rs2555104
rs11584821
rs17677991
rs7099229
rs6881568
rs11412296
rs76656449
rs13230646
rs17803849
rs116863223
rs7772289
rs7790856
rs6659669
rs1611236
rs2230590
rs145114957
rs6584579
rs113525195
rs80324517
rs6776756
rs8105767
rs12925933
rs117630647
rs76219171
rs45604339
rs7221585
rs12369950
rs3093888
rs16978028
rs61748181
rs869785
rs77231040
rs2977608
rs10112752
rs9398196
rs12412214
rs28502153
rs73730598
rs11117354
rs9878436
rs111527438
rs4731541
rs12451892
rs10905255
rs7209057
rs11426156
rs4498805
rs17445108
rs450962
rs11212631
rs9955360
rs4743037
rs73581419
rs117407747
rs11579626
rs376641875
rs2282764
rs59409453
rs11769630
rs12932179
A8
rs66731853
rs79977579
rs10840270
rs142730696
rs112394943
rs202034370
rs188918174
rs4616688
rs117034449
rs117512405
rs41269079
rs5742915
rs2538745
rs150150565
rs77732866
rs61405042
rs35640778
rs6587577
rs8102497
rs871134
rs10773176
rs6751209
rs34550383
rs142426306
rs10774624
rs11699829
rs4695407
rs2276182
rs1023767
rs7164950
rs139669835
rs10905346
rs141214782
rs111950327
rs4530278
rs1003322
rs3767952
rs131797
rs6007020
rs13062095
rs10845387
rs78491606
rs137901416
rs939916
rs2763979
rs3785074
rs6669563
rs9940099
rs7705526
rs28363070
rs80116508
rs1957937
rs932002
rs6536702
rs144204502
rs1332941
rs3891167
rs75664430
rs76065543
rs4724
rs11557154
rs143190905
rs35446936
SNP
0.000
0.025
0.050
0.075
Effect size (b)
